# A Case Report of the Triple Procedure in a Patient - Penetrating Keratoplasty, Extracapsular Cataract Extraction, and Posterior Chamber Intraocular Lens Implantation

**DOI:** 10.7759/cureus.28673

**Published:** 2022-09-01

**Authors:** Ronak Rukhiyana, Sachin Daigavane

**Affiliations:** 1 Department of Ophthalmology, Jawaharlal Nehru Medical College, Datta Meghe Institute of Medical Sciences University, Wardha, IND; 2 Department Of Ophthalmology, Jawaharlal Nehru Medical College, Datta Meghe Institute of Medical Sciences University, Wardha, IND

**Keywords:** capsulotomy, posterior chamber intraocular lens implantation, extracapsular cataract extraction, penetrating keratoplasty, the triple procedure

## Abstract

The triple procedure, which includes penetrating keratoplasty (PKP), extracapsular cataract extraction (ECCE), and posterior chamber lens implantation (PCIOL), is a surgical preference and a powerful remedy for patients with corneal pathologies and cataracts. In the patient, the PCIOL is implanted into the capsular bag, and the usage of a closed anterior chamber technique is recommended. 10-zero nylon sutures are used in the ten to twelve o'clock positions, and the donor corneal button is added. Continuous curvilinear capsulorhexis (CCC) is accomplished with a capsulotomy inserted through the side port, and the PCIOL is inserted through an open anterior chamber route. This triple system for the remedy of corneal illnesses with cataracts seems to be possible and practical.

The following is a case of a 47-year-old patient suffering from a corneal ulcer with pseudocornea due to trauma by an insect in the left eye. He was referred to the department of ophthalmology with complaints of diminution of vision, gradual and progressive, which is associated with excessive watering of the eyes and redness.

## Introduction

In a triple corneal procedure, keratoplasty and cataract surgery are combined. Cataract surgery is frequently combined with anterior lamellar keratoplasty, endothelial keratoplasty, and conventional penetrating keratoplasty. This article examines recent breakthroughs and current trends in triple corneal surgeries. The preferred course of treatment is simultaneous penetrating keratoplasty with extracapsular cataract extraction and implantation of a posterior chamber lens in patients who present with both cataract and medically uncontrolled corneal disease [1]. Unless contraindicated, extracapsular cataract extraction procedures are presently the medical option of choice for practically all types of adult and pediatric cataracts. The posterior capsule is unharmed, but a sizeable part of the anterior capsule, the epithelial nucleus, and the cortex are eliminated. The surgical techniques for extracapsular cataract extraction (ECCE) presently in trend are conventional extracapsular cataract extraction (ECCE), manual small incision cataract surgery (SICS), phacoemulsification, and femtosecond laser-assisted cataract surgery [2].

## Case presentation

A 47-year-old male patient came to the ophthalmology outpatient department at the tertiary care hospital with a history of trauma to the left eye by an insect three months ago. The patient is a laborer at a brick factory who presented with complaints of trauma by an insect in his left eye, after which he experienced a diminution of vision which is gradual and progressive in nature and was associated with excessive watering of the eyes and redness. There were no associated systemic complaints. The patient has no history of hypertension, diabetes, syphilis, tuberculosis, or bronchial asthma. There is no significant family history as well.

The patient was cooperative and well-oriented with time, place, and person. He has an average build and is afebrile in nature. His pulse rate was 73 beats per minute, and blood pressure was recorded at 125/80 mmHg. There was no pallor, clubbing, icterus, or lymphadenopathy on examination. On examination, the central nervous and cardiovascular systems were within normal limits. The patient's chest was clear bilaterally, and on per abdominal examination, the patient had no apparent distress. The patient had no complaints of cough or fever. The general condition was moderate. The temperature, pulse, respiratory rate, and blood pressure were stable.

Left eye examination revealed visual acuity of perception of light positive and perception of rays accurate. Conjunctiva showed congestion, both superficial and deep. Pseudocornea was present with mild edema. Other intraocular details could not be appreciated. For pre-operative preparation, the refractive status of the eye was evaluated, and an intra ocular lens (IOL) was picked dependent on that. The Sanders-Retzlaff-Kraff equation was utilized to process the IOL power [3]. TThe patient was examined and a peribulbar anaesthesia was given. Subjectively, the intraocular pressure was measured by digital tonometry. The intraocular and orbital pressures were reduced when the eyes were anesthetized, improving the circumstances for the procedure. After the anesthesia was provided, the area was disinfected and sterilized.

**Figure 1:**
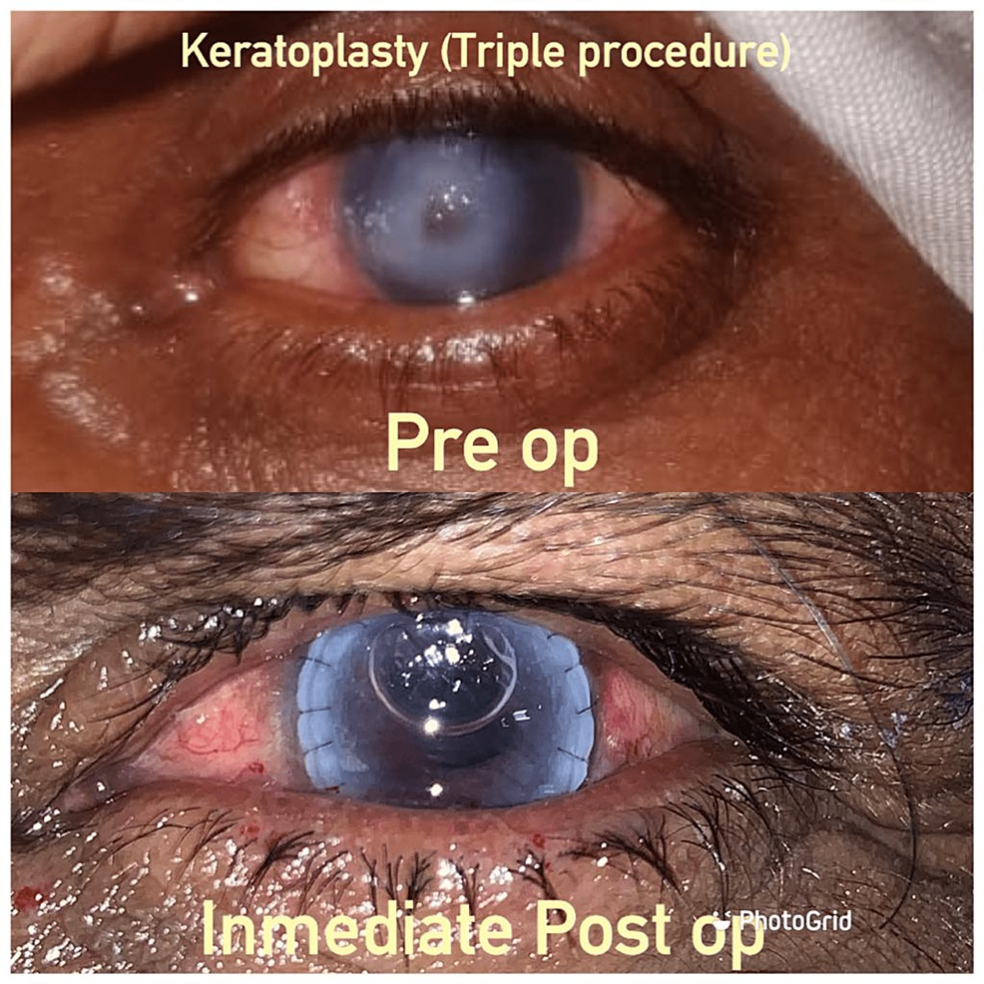
Pre operative and post operative keratoplasty

The patient was given effective antibiotic eye drops multiple times, day by day, fourteen days before the medical procedure, after the hazards of the careful activity were unveiled to them, and informed consent was procured. Under local anesthetic, a 7 mm entering corneal graft was done from the donor cornea. As shown in Figure 1, therapeutic penetrating keratoplasty was performed with placement of the graft; 17 sutures were taken, following which ECCE and posterior chamber lens implantation (PCIOL) were done simultaneously [4]. Sutures of 10/0 nylon were utilized to stitch the graft in a hindered or persistent way. These were liberated if there were any bonds in the middle of the iris, lens, and cornea. At the end of the surgery, a blend of antibiotics and steroids was infused into a lower most fornix. The eye was patched until epithelial healing was over [5]. Topical antibiotics, steroid drops, immunosuppressant high-frequency topical cyclosporine 0.05%, lubricating eyedrops, and sodium chloride eyedrops with a brief duration of action were used postoperatively. We used systemic drugs, analgesics, and anti-inflammatory medicines as needed during the patient's presence in the hospital.

The patient was kept on regular follow-up. Symptomatically, improvement was seen. Treatment was strictly monitored. The patient was advised about the treatment. During monthly follow-up, we recorded uncorrected visual acuity and intraocular pressure using an applanation tonometer. On his two-month follow-up, a best corrected visual acuity of 6/24p was recorded on the patient. He was continued on treatment and kept on strict follow-up for careful monitoring of chances of graft failure.

## Discussion

The triple procedure has become a grounded and viable careful therapy for patients with lenticular and corneal opacity [6]. Single step triple procedure decreases the patient's clinical stay and post-operative care, particularly in old patients who ordinarily have geriatric medical conditions. Triple surgery gives quicker visual recovery and is more liked than consecutive or two-staged procedures [7]. Some time ago, a test of intraocular lens power computation for triple surgery existed because of the corneal scar. Yet, at this point, it is usually calculated by the utilization of normal consistent keratometry of 44 D, as well as individual eye keratometry is additionally a choice [8]. In two-stage or consecutive medical procedures, we get more precise intraocular lens power after removing stitches of penetrating keratoplasty.

Penetrating keratoplasty speeds up cataract development, especially in eyes with pre-existing moderate cataracts, because of the meticulous injury and aggravation, as well as the post-surgical effective steroid treatment [9]. In consecutive or two-stage medical procedures, there might be endothelial cell degradation from the tension produced during cataract medical procedure, including low endothelial cell count, and corneal decompensation may happen; accordingly, the triple stage surgery is the defended choice so as to obtain a tremendous visual result for similar patients [10]. Corneal graft, which is evident following the triple surgery, may go up to 60% to 100% in accuracy. Signs of corneal transplantation significantly affect graft endurance, and the patient's determination is very useful for the accomplishment of the medical procedure.

Epithelial imperfections, two-degree glaucoma, corneal vascularization, decentration of the posterior chamber intraocular lens, as well as graft failure are familiar problems of triple surgery [6]. Better visual acuity managed intraocular stress, and clean charts unaccompanied by edema in triple surgical operation became discovered better with a continous curvilinear capsulorhexis than can opener capsulotomy [11]. Accomplishment for this situation might be because of the patient selected, little operative interruption, utilization of better donor cornea, viscoelastic material, continuous curvilinear capsulorrhexis as well as great intraocular lens placement in the bag, legitimate assessment of intraocular lens power, and standard development with slit lamp assessment which allowed for appropriate and timely post-useful intercession, such as YAG capsulotomy [12].

## Conclusions

This is a case report of triple surgery involving penetrating keratoplasty, extracapsular cataract extraction, and posterior chamber intraocular lens implantation to treat corneal diseases and cataracts, which is simple, effective, and gives an excellent result as well as uninterrupted vision and better visual recovery. The triple procedure is a successful surgical approach for corneal problems linked to cataracts. It offers the best possible visual and refractive results, particularly in high-risk graft settings. This surgery should be preferred over consecutive procedures or two-staged procedures.
